# What Caused the UK's Largest Common Dolphin (*Delphinus delphis*) Mass Stranding Event?

**DOI:** 10.1371/journal.pone.0060953

**Published:** 2013-04-30

**Authors:** Paul D. Jepson, Robert Deaville, Karina Acevedo-Whitehouse, James Barnett, Andrew Brownlow, Robert L. Brownell Jr., Frances C. Clare, Nick Davison, Robin J. Law, Jan Loveridge, Shaheed K. Macgregor, Steven Morris, Sinéad Murphy, Rod Penrose, Matthew W. Perkins, Eunice Pinn, Henrike Seibel, Ursula Siebert, Eva Sierra, Victor Simpson, Mark L. Tasker, Nick Tregenza, Andrew A. Cunningham, Antonio Fernández

**Affiliations:** 1 Institute of Zoology, Zoological Society of London, Regent's Park, London, United Kingdom; 2 Unit for Basic and Applied Microbiology, School of Natural Sciences, Autonomous University of Queretaro, Queretaro, Mexico; 3 Animal Health and Veterinary Laboratories Agency Truro, Polwhele, Truro, Cornwall, United Kingdom; 4 Scottish Agricultural College, Inverness, United Kingdom; 5 NOAA Fisheries, Southwest Fisheries Science Center, Pacific Grove, California, United States of America; 6 Centre for Environment, Fisheries and Aquaculture Science, Lowestoft, United Kingdom; 7 Cornwall Wildlife Trust Marine Strandings Network, Five Acres, Allet, Truro, United Kingdom; 8 Centre for Environment, Fisheries and Aquaculture Science, Weymouth, Dorset, United Kingdom; 9 Marine Environmental Monitoring, Penwalk, Llechryd, Cardigan, Ceredigion, United Kingdom; 10 Joint Nature Conservation Committee, Aberdeen, United Kingdom; 11 Institute of Terrestrial and Aquatic Wildlife Research, University of Veterinary Medicine Hannover, Büsum, Germany; 12 Histology and Pathology Unit, Institute for Animal Health, Veterinary School Montana Cardones-Arucas, University of Las Palmas de Gran Canaria, Gran Canaria-Spain; 13 Wildlife Veterinary Investigation Centre, Chacewater, Truro, Cornwall, United Kingdom; Texas A & M University-Corpus Christi, United States of America

## Abstract

On 9 June 2008, the UK's largest mass stranding event (MSE) of short-beaked common dolphins (*Delphinus delphis*) occurred in Falmouth Bay, Cornwall. At least 26 dolphins died, and a similar number was refloated/herded back to sea. On necropsy, all dolphins were in good nutritive status with empty stomachs and no evidence of known infectious disease or acute physical injury. Auditory tissues were grossly normal (26/26) but had microscopic haemorrhages (5/5) and mild otitis media (1/5) in the freshest cases. Five lactating adult dolphins, one immature male, and one immature female tested were free of harmful algal toxins and had low chemical pollutant levels. Pathological evidence of mud/seawater inhalation (11/26), local tide cycle, and the relative lack of renal myoglobinuria (26/26) suggested MSE onset on a rising tide between 06∶30 and 08∶21 hrs (9 June). Potential causes excluded or considered highly unlikely included infectious disease, gas/fat embolism, boat strike, by-catch, predator attack, foraging unusually close to shore, chemical or algal toxin exposure, abnormal weather/climatic conditions, and high-intensity acoustic inputs from seismic airgun arrays or natural sources (e.g., earthquakes). International naval exercises did occur in close proximity to the MSE with the most intense part of the exercises (including mid-frequency sonars) occurring four days before the MSE and resuming with helicopter exercises on the morning of the MSE. The MSE may therefore have been a “two-stage process” where a group of normally pelagic dolphins entered Falmouth Bay and, after 3–4 days in/around the Bay, a second acoustic/disturbance event occurred causing them to strand *en masse*. This spatial and temporal association with the MSE, previous associations between naval activities and cetacean MSEs, and an absence of other identifiable factors known to cause cetacean MSEs, indicates naval activity to be the most probable cause of the Falmouth Bay MSE.

## Introduction

Cetacean mass stranding events (MSEs) are commonly described as two or more cetaceans (excluding a cow-calf pair) of the same species coming ashore, usually alive, at the same time and place [1]. Historically, at least 19 cetacean species have been affected, with some species, including false killer whales (*Pseudorca crassidens*), long-finned pilot whales (*Globicephala melas*), short-finned pilot whales (*Globicephala macrorhynchus*), Atlantic white-sided dolphins (*Lagenorhynchus acutus*) and white beaked dolphins (*Lagenorhynchus albirostris*), stranding more often than others [1], [2]. The cause of these strandings has puzzled humans for centuries and, although numerous theories have been developed, few MSEs have been studied in detail and seldom has a definitive cause been established (reviewed in [1]).

Cetaceans that mass strand are generally pelagic odontocetes with a highly evolved social structure (reviewed in [1]). Proposed causal factors for cetacean MSEs are numerous and include: becoming trapped on a receding tide (e.g. in long meandering channels, broad tidal flats, strong or unusual currents, extreme tidal flow or volume) [3]; navigational errors associated with topographical features forming natural “whale traps” (such as Wellfleet Bay, Cape Cod, Massachusetts, USA; [4]); geomagnetic disturbances and errors in navigation while following geomagnetic contours [5]; disturbance of echolocation by multiple reflections in bays [6]; pelagic cetaceans following prey close inshore [7]; escaping from predators [1]; disease in one or more individuals in a social group leading to some or all of the remainder of the group stranding [8]; algal toxins and unusual environmental conditions such as electrical storms and other meteorological events; earthquakes and high-intensity acoustic fields (reviewed in [1]). Species-specific behaviour in response to “panic” is also suggested as a potential factor explaining why some cetacean species mass strand more frequently than others (reviewed in [1]).

Prior to the common dolphin (*Delphinus delphis*) MSE in Falmouth Bay on 9 June 2008, only three common dolphin MSEs, all of undetermined cause, have been recorded in the UK. One of these consisted of five dolphins on 3 November 1915 at Tean, Isles of Scilly; another, of five animals near Invergordon, Ross and Cromarty on 8 February 1937, and a third involved 15 dolphins near Pembroke, Wales on 11 August 1938 (listed by [9]). Between1990–2011, 1,714 common dolphins were reported stranded in the UK and 976 in Cornwall (data: Cornwall Wildlife Trust Marine Strandings Network, UK). In the same period over 500 systematic post-mortem examinations have been conducted on UK-stranded common dolphins (over 300 of them from Cornwall), with the majority (approx. 51%) determined to have died due to incidental capture in commercial fishing gear (by-catch) [10], [11], [12]. By-catch events, in which dead stranded animals are widely distributed, are very different spatially and temporally from the MSE reported in the present study.

Three common dolphin MSEs have occurred since 2001 along the Irish and French coastlines that were studied. In February 2001, 15 common dolphins live stranded on the Mullet Peninsula, west coast of Ireland [13] and a group of six common dolphins live stranded in May 2002 in Ballyvaughan, Co. Clare on the west coast of Ireland [13], [14]. The French MSE involved approximately 100 individuals at Pleubian, Brittany in February 2002 [15], [16]. The causes of these MSEs were never ascertained, although in the case of the Mullet Peninsula event and two previous common dolphin MSEs on this Peninsula in 1998 and 1999, the topography of the area, presence of ever changing sand banks and exceptionally low tides all might have influenced the MSEs [3].

**Figure 1:**
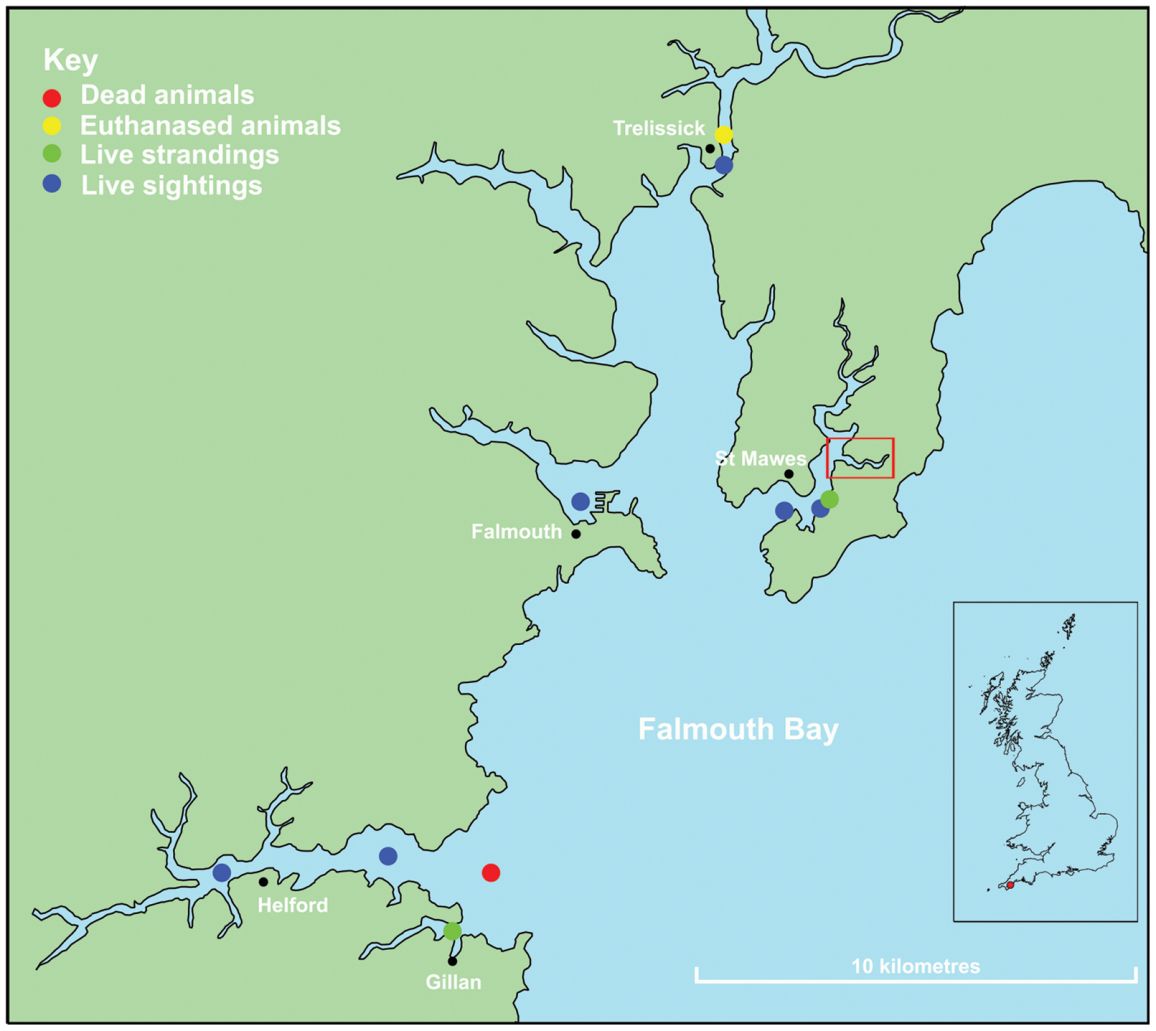
Locations of live sightings/strandings and dead/euthanized common dolphins in Falmouth Bay, Cornwall on 9 June 2008 (inset map shows location of Falmouth Bay in UK). Red box show location of 25/26 dead and necropsied animals found in Porth Creek, Cornwall (see Figure 2). Data sourced from Cornwall Wildlife Trust Marine Strandings Network.

The Falmouth Bay Mass Stranding Event (9 June 2008). At 08∶21 hrs (BST) on 9 June 2008, an initial report was received by Falmouth Marine Coastguard Agency of a dead short-beaked common dolphin in Porth Creek, a small tributary of the Percuil River. The Percuil River is a large tributary of the Fal Estuary, a deep tidal ria in Cornwall, in the southwest of the UK that has a tidal range of around 5 m (see **Figures** **1** **and** **2**). Subsequently, additional live and dead dolphins were found in the area and trained volunteers worked during the day on foot, in boats and swimming, to recover more carcasses and to try to herd larger groups of live common dolphins back out to sea. The earliest eyewitness report of a live dolphin stranding was at Cellar Beach in the village of Place at approximately 08∶15 but the exact timing of this sighting is unconfirmed (**Table** **1** **,** **Figure** **1**). This live dolphin was refloated twice and later joined a larger free-swimming dolphin group. The total number of dolphins involved in the MSE is not definitely known, but 24 common dolphins were eventually found dead in Porth Creek on 9 June and another two that stranded alive (one in Porth Creek and one near Trelissick approx. 6.5 km NNW of Porth Creek in the River Fal) were euthanized. All of the dead dolphins found in Porth Creek were below the high water level (some closer to mid-water) and all were retrieved for necropsy. A dead dolphin was reported floating at the mouth of the Helford River on 11 June but this was not confirmed and no carcass was obtained for necropsy. The stranding locations on 9 June covered approximately 20 km of the Falmouth Bay and estuary coastline from Gillan Creek to Trelissick (**Figures** **1** **and** **2**).

**Figure 2:**
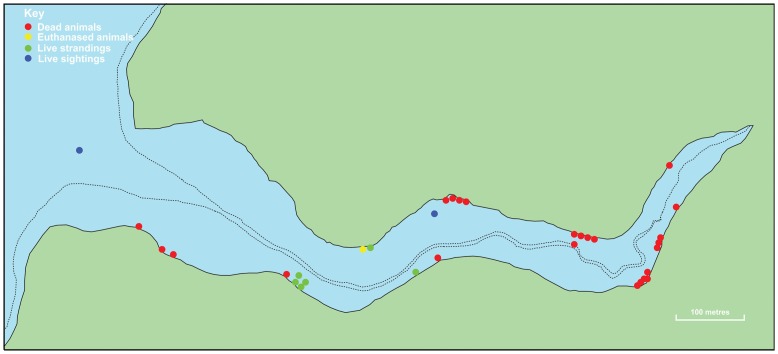
Locations of live sightings/strandings and dead/euthanized common dolphins found in Porth Creek, Percuil River, Cornwall on 9 June 2008. Solid line  =  high water mark. Dotted line  =  low water mark. Data sourced from Cornwall Wildlife Trust Marine Strandings Network.

**Table 1:** Live sightings of common (*D. delphis*) and bottlenose dolphins (*T. truncatus*) in or near Falmouth Bay, Cornwall (5–9 June 2008).

DATE	SPECIES	LOCATION	GROUP SIZE	TIME	NOTES	OBSERVER/SOURCE
5 June	*D. delphis*	Porthoustock, Falmouth Bay	50	N/A	½ mile east of Porthoustock. Lots of activity – gannets diving	Orca Seasafaris
5 June	*D. delphis*	Off Port Mellon, Chapel Point	30	N/A	/	CWTMSN
5 June	*D. delphis*	Mevagissey (E of Falmouth Bay)	40	N/A	Pod had been feeding off Polstreath and were heading SE toward Chapel Point	Marie Pearce
5 June	*D. delphis*	Off Penzer Point (W of Mousehole), Mount's Bay	5	14.31	Feeding and playing around boat	Duncan Jones Marine Discovery
7 June	*D. delphis*	Off The Manacles, Falmouth Bay	30–40	14.02	200 metres off coastline	Orca Seasafaris
7 June	*D. delphis*	Helford River, Falmouth Bay	2	11.30	Free swimming	CWTMSN
7 June	*D. delphis*	Mouth of Helford River, Falmouth Bay	2	15.10	Free swimming	CWTMSN
7 June	*T. truncatus*	Hand Deeps Reef, off South coast Cornwall	4+	N/A	Feeding around fishing boat	CWTMSN
8 June	*D. delphis*	Off Portscatho, Cornwall	50–60	14.30	Free swimming about 0.25 miles or less offshore	CWTMSN
8 June	*T. truncatus*	Falmouth Bay, Cornwall	2	17.22	/	Royal Navy MMO
9 June	*D. delphis*	Cellars Beach, Place (mouth of the Percuil River), Cornwall	1	08.15 (approx)	One dolphin stranded alive (later refloated and joined larger dolphin group).	BDMLR/CWTMSN
9 June	*D. delphis*	Porth Creek	1	08:21	One dead dolphin reported to HM Coastguard in Porth Creek	Falmouth Coastguard
9 June	*D. delphis*	Porth Creek, Cornwall	7	AM	Rescued and returned to deep water by BDMLR and others. (25 other common dolphins died/euthanized in Porth Creek)	BDMLR/CWTMSN
9 June	*D. delphis*	Porth Creek	1	AM	Live-stranded, euthanized	BDMLR/CWTMSN
9 June	*D. delphis*	Mouth of Porth Creek	20–30	AM	Free swimming, milling behaviour^1^, later herded out to sea	BDMLR/CWTMSN
9 June	*D. delphis*	Place, mouth of Percuil River, Cornwall	35–40	AM	Free swimming, milling behaviour^1^, later herded out to sea	BDMLR/CWTMSN
9 June	*D. delphis*	Inner part of Falmouth Harbour, Cornwall (approx 5km due west from Porth Creek)	15	09.30	Free swimming, milling behaviour^1^, two attempted to strand, ignored boats/humans, resisted attempts to shepherd them out to sea. Dolphins later swum back to sea overnight.	BDMLR/CWTMSN
9 June	*D. delphis*	Gillan Creek, Falmouth Bay, Cornwall	12	16.30	Free swimming, milling behaviour^1^, seven attempted to strand, herded back to sea	BDMLR/CWTMSN
9 June	*D. delphis*	Trelissick (approx. 6.5km NNW of Porth Creek) in the River Fal	1	16.30	Live-stranded, euthanized	BDMLR/CWTMSN
11 June	*T. truncatus*	Mounts Bay, Cornwall	2	12.18	Playing around a wildlife tour boat	CWTMSN

1-“ milling” is typical pre-stranding behaviour as described by [1]. This includes a single, cohesive pod swimming in tight circles accompanied by frequent behaviours such as spy hopping, fluke slapping, and audible vocalizations.

BDMLR  =  British Divers Marine Life Rescue CWTMSN  =  Cornwall Wildlife Trust Marine Strandings Network.

Groups of free-swimming common dolphins were also reported to be seen in Falmouth Harbour at 09∶30 hrs and Gillan Creek at 16∶30 hrs on 9 June and at the mouth of Porth Creek and near Place through the morning of 9 June (see **Figures** **1** **and** **2** and **Table** **1**). Eyewitnesses consistently described their behaviour as “swimming continuously in tight circles, being vocal, fluke slapping, leaning sideways, and often with one or more individuals attempting to strand”. The dolphins in Falmouth Harbour were also described by eyewitnesses as “appearing to ignore boats and the presence of humans and were resistant to attempts to herd them out of the harbour” [17]. An unknown number of dolphins was successfully guided into the main estuary and swam towards conspecific(s) further out in deeper water. It is not known whether any of the dolphins that were seen free-swimming close to shore on the morning of 9 June at Place and Porth Creek were any of those seen later that morning in Falmouth Harbour and that afternoon at Gillan Creek and Trelissick. High water in Porth Creek on 8 June was at 09∶44 and 21∶52 hrs and on 9 June at 10∶37 hrs (BST). The dolphins furthest into the creek were at a point where the only water present at low tide is a shallow stream. The weather on the day of the MSE and on the days immediately prior to the event was dry and sunny with no unusual storm activity in the region (data: UK Met Office).

The objectives of the present study were to conduct pathological investigations on the individuals that died and to investigate all available evidence to determine possible causes of the MSE.

## Materials and Methods

### Information on live and dead dolphins involved in the MSE

Information on the times of discovery and locations of live and dead-stranded common dolphins involved in the MSE was gathered from a variety of opportunistic sources. Volunteers from Cornwall Wildlife Trust Marine Strandings Network (CWTMSN) and British Divers Marine Life Rescue (BDMLR) were interviewed after the event and further information was solicited from bystanders and local maritime agencies including the Coastguard, the Falmouth Harbour Authority and the Cornwall Sea Fisheries Committee. Anecdotal reports from eyewitnesses of the events prior to, during and after the MSE were examined to try to clarify the timing and significance of any activities that may have had a bearing on the event. Collated information is shown in Figs. 1 and 2 and Table 1.

### Gross necropsies

Carcasses were recovered from the intertidal zone and all 26 dead common dolphins were systematically examined using a standard necropsy protocol [11] beginning within 8 hours of being found dead and terminated 3 days later. Ambient temperature was 14–25°C on the day of stranding. Sexual maturity was determined from gonadal material. Five-ten teeth were obtained from the middle of the left mandible for age estimation and stored frozen (−20°C). Six ears (tympanoperiotic complexes) from the freshest dolphins (Table 2) were grossly dissected and preserved in 10% formalin for further examination. A standard set of tissue samples was taken, depending on carcass condition, for a range of standard diagnostic tests including microbiology, histopathology, immunohistochemistry (anti-fibrinogen and anti-myoglobin), molecular detection of morbillivirus and other diagnostic studies (see below).

**Table 2:** Pathological and other data for 26 common dolphins examined at necropsy.

Reference Number	Sex	Age (yrs)	Age class	State of decomp. (on finding)	State of decomp. (on necropsy)	Food in stomach	Significant pre-existing disease	Evidence of trauma (eg bycatch)	Mud/water in lungs	Ears (gross exam)	Ears (histopath.)	Brucella sp. infection	Gas/fat emboli	Morbillivirus infection	Algal toxins (liver)	Chemical toxins
SW2008/94.1	M	3	Juv.	Fresh	Slight	negative	negative	negative	YES	NAD	not examined	negative	negative	negative	not tested	not tested
SW2008/94.2	F	3	Juv.	Fresh	Fresh	negative	negative	negative	NO	NAD	Left only	negative	negative	negative	not tested	not tested
SW2008/94.3	F	3	Juv.	Fresh	Fresh	negative	negative	negative	YES	NAD	not examined	negative	negative	negative	not tested	not tested
SW2008/94.4	F	5	Juv.	Fresh	Fresh	negative	negative	negative	YES	NAD	not examined	negative	negative	negative	not tested	not tested
SW2008/94.5	F	9	Adult	Fresh	Slight	negative	negative	negative	TRACE	NAD	not examined	negative	negative	negative	negative	tested*
SW2008/94.6	M	9	Juv.	Fresh	Slight	negative	negative	negative	YES	NAD	not examined	negative	negative	negative	negative	tested*
SW2008/94.7	F	>20	Adult	Fresh	Moderate	negative	negative	negative	NO	NAD	not examined	negative	negative	negative	negative	tested*
SW2008/94.8	F	>16	Adult	Fresh	Slight	negative	negative	negative	YES	NAD	not examined	negative	negative	negative	negative	tested*
SW2008/94.9	M	1.9	Juv.	Fresh	Fresh	negative	negative	negative	NO	NAD	not examined	negative	negative	negative	not tested	not tested
SW2008/94.10	M	1.9	Juv.	Fresh	Fresh	negative	negative	negative	NO	NAD	Left and Right	negative	negative	negative	not tested	not tested
SW2008/94.11	F	8	Juv.	Fresh	Slight	negative	negative	negative	NO	NAD	not examined	negative	negative	negative	not tested	not tested
SW2008/94.12	F	25	Adult	Fresh	Fresh	negative	negative	negative	NO	NAD	Right only	negative	negative	negative	negative	tested*
SW2008/94.13	F	11	Adult	Fresh	Fresh-slight	negative	negative	negative	NO	NAD	not examined	negative	negative	negative	negative	tested*
SW2008/94.14	F	8	Juv.	Fresh	Fresh	negative	negative	negative	YES	NAD	Left and Right	negative	negative	negative	not tested	not tested
SW2008/94.15	F	3	Juv.	Fresh	Fresh	negative	negative	negative	YES	NAD	not examined	negative	negative	negative	not tested	not tested
SW2008/94.16	M	2	Juv.	Fresh	Slight	negative	negative	negative	YES	NAD	not examined	negative	negative	negative	not tested	not tested
SW2008/94.17	F	3	Juv.	Fresh	Moderate	negative	negative	negative	NO	NAD	not examined	negative	negative	negative	not tested	not tested
SW2008/94.18	M	1.9	Juv.	Fresh	Slight-mod.	negative	negative	negative	NO	NAD	not examined	negative	negative	negative	not tested	not tested
SW2008/94.19	M	2	Juv.	Fresh	Fresh	negative	negative	negative	YES	NAD	not examined	negative	negative	negative	not tested	not tested
SW2008/94.20	M	4	Juv.	Fresh	Slight	negative	negative	negative	YES	NAD	not examined	negative	negative	negative	not tested	not tested
SW2008/94.21	M	3	Juv.	Fresh	Moderate	negative	negative	negative	TRACE	NAD	not examined	negative	negative	negative	not tested	not tested
SW2008/94.22	F	4.9	Juv.	Fresh	Slight	negative	negative	negative	YES	NAD	not examined	negative	negative	negative	negative	tested*
SW2008/94.23	M	3	Juv.	Fresh	Fresh-slight	negative	negative	negative	NO	NAD	not examined	negative	negative	negative	not tested	not tested
SW2008/94.24	M	1.9	Juv.	Fresh	Moderate	negative	negative	negative	NO	NAD	not examined	negative	negative	negative	not tested	not tested
SW2008/94.25	M	N/D	Juv.	Fresh	Moderate-advanced	negative	negative	negative	TRACE	NAD	not examined	negative	negative	negative	not tested	not tested
SW2008/95	M	N/D	Juv.	Fresh	Fresh	negative	negative	negative	NO	NAD	not examined	Brucella ceti (testis only)	negative	negative	not tested	not tested

N.B. All 26 dead dolphins were in good nutritive condition when found on 9 June 2008 NAD  =  No abnormalities detected. N/D  =  No data.

* - see “Chemical and algal toxin detection” (Results section) for further information.

### Bacteriology

Tissue samples or swabs of selected tissues, including liver, kidney, lung and brain, were taken aseptically for bacteriological examination using aerobic, anaerobic and capnophilic incubation and standardised methods [11]. Swabs or tissues were inoculated directly onto either Columbia blood agar base (Oxoid, CM331) with 5% horse blood and incubated aerobically, anaerobically or capnophilically at 37°C and observed at 1, 2 and 5 days, or inoculated onto 5% sheep blood agar (Oxoid, CM0271) and MacConkey agar (Oxoid CM0007) and incubated at 37°C in a capnophilic atmosphere and examined daily for 7 days. Lung samples were incubated aerobically on Columbia blood agar base (Oxoid, CM331) with 5% horse blood and on Sabourauds dextrose agar (Oxoid CM41) in an aerobic atmosphere for fungal isolation. Any organisms recovered were identified using conventional methods including growth characteristics, colony morphology, staining properties and biochemical characterisation using the API identification system (bioMérieux, France). Culture methods and identification of *Brucella* species isolated from tissues utilised standardised methodologies similar to those described by [18] and were confirmed as *Brucella ceti* using (i) Polymerase Chain Reaction (PCR) amplification of an IS*711* element downstream of the base-pair (bp) 26 gene, (ii) PCR amplification of the outer membrane proteins (*omp*) 2 locus and (iii) analysis of the genome using restriction enzymes [19], [20].

### Histopathology

A range of tissue samples from all 26 dead dolphins was preserved in neutral buffered 10% formalin, embedded in paraffin, sectioned at 2–6 µm and stained with haematoxylin and eosin (H&E) for histological examination according to standardized protocol [11]. Five tympano-periotic complexes and one cochlea were investigated microscopically at the Institute of Terrestrial and Aquatic Wildlife Research in Büsum, Germany using a similar protocol to that used to examine UK-stranded harbour porpoises [21] and based mainly on methods described by [22]. Briefly, decalcification was conducted by immersion in 0.27 M EDTA (disodium ethylenediaminetetraacetate) at room temperature. The decalcification solution also contained 1% formalin and was changed weekly. Complete decalcification was determined by X-ray radiography. All specimens were then neutralised in tap water for 24 hours, then in distilled water for 4 to 6 hours followed by progressive dehydration with 50%/70%/80%/95%/100% alcohol. The last two solutions (95% and 100%) were changed twice before the samples were finally placed into ether-alcohol (1∶1). The ear tissues were then embedded in 1.5% celloidin for a week, 3% celloidin for another three weeks, then 6% celloidin for three to four weeks and finally into 12% celloidin for another three to four weeks. The formed celloidin-block was hardened with chloroform in a desiccator at 4°C for two weeks, after which the celloidin was hardened with fresh chloroform for another two weeks. At this stage the block was trimmed and set in cedarwood oil for at least one week then set into fresh cedarwood oil for another week. The blocks were then sectioned (20 µm thick sections) using a microtome. Every 10th section was stained with H&E and mounted while the rest of the tissue sections were stored in 80% alcohol. Around one hundred stained slides per ear were examined microscopically.

### Immunohistochemistry

Sections of formalin-fixed lung (n = 25) and mesenteric/pulmonary associated lymph node (n = 26) were processed by sectioning and staining with osmium-tetroxide post-fixation followed by embedding in paraffin using standard methods [23] to demonstrate fat emboli. Immunohistochemical techniques to demonstrate fibrinogen in skeletal and cardiac muscle and myoglobin in kidney were identical to those described by [24]. As a positive control for myoglobin in cetacean kidney, renal sections were used from a striped dolphin (*Stenella coeruleoalba*) with stranding (capture) myopathy associated with prolonged beach stranding and in which myoglobinuria had been reported by [24].

### Assessment of sexual maturity and age

Females were classified as sexually mature if, on gross examination, one or more ovarian corpora scars were present [25]. Samples of testicular tissue were processed for histology and males were classified as mature if testicular tissue showed evidence of spermatogenesis, i.e. spermatozoa were present (after [26]). The straightest and least worn teeth were selected for age estimation using the wax embedding technique outlined in [27]. This consisted of fixing teeth in 10% neutral buffered formalin for at least two weeks, decalcifying using the rapid decalcifier RDO©, and using standard histological processing techniques teeth were dehydrated, embedded in paraffin wax, sectioned at 5 μm using a microtome and stained using 60% Harris's haematoxylin.

### Morbillivirus detection

Total RNA was extracted from sections of frozen (−80°C) lung (n = 26) and brain (n = 22) samples and the presence of morbilliviral RNA was tested using reverse transcriptase polymerase chain reaction targeting the conserved N terminus of the morbillivirus N gene [28]. All reactions were conducted in duplicate.

### Analysis of chemical toxins

All tissue samples were collected using standard methodology and stored at –20°C prior to preparation and analysis. Frozen liver samples were analysed for a range of trace elements (Cr, Ni, Cu, Zn, As, Se, Mn, Fe, Ag, Cd, Hg and Pb) and butyltins (monobutyltin, dibutyltin and tributyltin) (mg/kg wet weight). The hepatic molar Hg:Se ratio was also calculated [11], [29], [30]. Wet weight concentrations (mg/kg) of 25 individual chlorobiphenyl congeners (IUPAC numbers: 18, 28, 31, 44, 47, 49, 52, 66, 101, 105, 110, 118, 128, 138, 141, 149, 151, 153, 156, 158, 170, 180, 183, 187, 194) and a range of organochlorine pesticides and metabolites were determined in blubber samples according to previously established and validated protocols using internationally standardised methodologies (see [11], [29], [30], [31]). The sum of the concentrations of the 25 CB congeners (Σ25CBs) and organochlorine pesticides tested were determined and were then converted to a lipid basis (mg kg^−1^ lipid) using the proportion of hexane extractable lipid (%HEL) in individual blubber samples. For all analyses, appropriate quality control materials (certified or laboratory reference materials) were analysed within each sample batch in order that the day-to-day performance of the methods could be monitored.

### Analysis of algal toxins

Frozen samples (−80°C) of liver were analysed for the presence of harmful marine algal toxins using high performance liquid chromatographic techniques deployed for the purpose of toxin monitoring in commercial shellfish. For amnesic and paralytic shellfish poisoning toxin groups (ASP and PSP), liquid chromatographic (LC) methods were applied with photodiode array detection [for ASP see [32]] and fluorescence detection [for PSP see [33] and [34]]. Liquid chromatography with tandem mass spectrometric (MS/MS) detection was also used to confirm any analytical observations from these initial analyses (for ASP; [35]). For the diarrhetic shellfish poisoning (DSP) toxins as well as other, co-extracted lipophilic toxins, a LC-MS/MS method was also applied following [36]. The range of ASP toxins included domoic acid (DA) and its associated isomers – *epi*-DA, *iso*DA-A, *iso*DA-D and *iso*DA-E. The targeted toxins of the DSP were okadaic acid (OA), the dinophysistoxins (DTX1, DTX2 and acyl esters of OA and DTXs), pectenotoxins 1, 2 and 11, azaspiracids (AZA1, AZA2 and AZA3), yessotoxin (YTX) compounds - YTX, *homo* YTX, 45 OH YTX and 45 OH *homo* YTX and representatives of the cyclic imine groups – 13-desmethyl spirolide C (SPX1) and gymnodimine. For the PSP group, the following toxins were included – saxitoxin (STX) and its derivatives neosaxitoxin (NEO), gonyautoxins (GTX) 1 to 5, decarbamoyl (dc)STX, dcGTX2,3 and the *N*-sulphocarbamoyl gonyautoxins –2 and –3 (C1 and C2) toxins.

## Results

**Figure 3:**
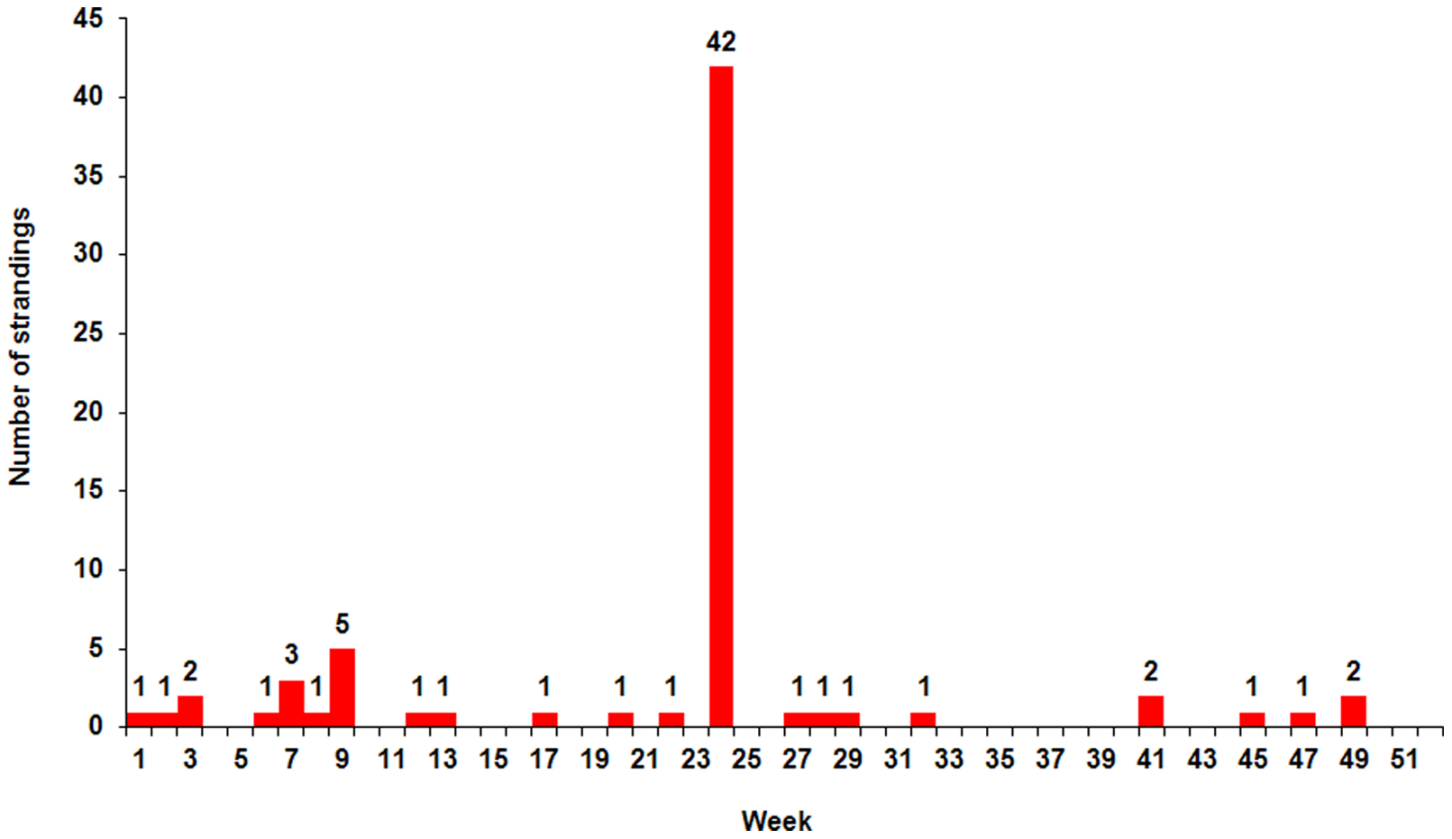
All common dolphin strandings (live and dead) in Cornwall (by week) Jan-Dec 2008 (Data: CWT Marine Strandings Network).

The locations of the live and dead dolphins reported in this MSE are shown in Figs. 1 and 2. The temporal distribution of all common dolphin strandings recorded in Cornwall throughout 2008 is shown in Fig. 3.

### Gross and histopathological examinations

Twenty-six common dolphin carcasses composed of 13 sexually immature males, 8 sexually immature females and 5 sexually mature females) were retrieved from the MSE for necropsy (Table 2). Individuals ranged in age from 2 to 25 yrs (males 2 to 9 yrs, and females from 3 to 25 yrs). All 26 dead dolphins found on 9 June were in freshly dead condition when initially recovered. The time between death and necropsy varied and not all carcasses could be refrigerated, so the state of decomposition at necropsy was fresh (n = 10); fresh-slight decomposition (n = 2); slight decomposition (n = 8); slight-moderate decomposition (n = 1); moderate decomposition (n = 4) and moderate-advanced decomposition (n = 1). The main gross and microscopic findings were similar in all cases (Table 2). All dolphins appeared to be in good nutritional condition and showed no significant evidence of acute physical injury or disease. No acute traumatic lesions characteristic of by-catch [12], boat impact [11] or bottlenose dolphin attack [11], [37], [38] were seen in any of the dead (or surviving) common dolphins. One dolphin had a chronic but relatively minor (old) injury associated with granulation tissue and localised fibrosis on the rostral tip of the maxillae (upper beak). Low intensity parasitic infestations were typical, most frequently in the lungs, and these were associated with relatively mild host tissue reactions which are commonly found in stranded common dolphins in UK waters [11], [12]. The stomachs were free of recently-ingested prey in all cases. Gonadal tissue was grossly and histologically normal in all 26 animals. However, three immature females (SW2008/94.3, SW2008/94.4, SW2008/94.15) ranging from 3 to 5 years in age had colostrum-type fluid present in their mammary glands. All five adult females were actively lactating. A small (1–2 cm) raised, pale, plaque-like like lesion was found on the dorsal aspect of the penis in one immature male common dolphin, 1.8 years of age (SW2008/94.18). Histopathology of this lesion confirmed focal thickening of the epithelium (resembling a plaque) without associated inflammatory response or presence of inclusion bodies, consistent with viral (papilloma virus) infection.

**Figure 4:**
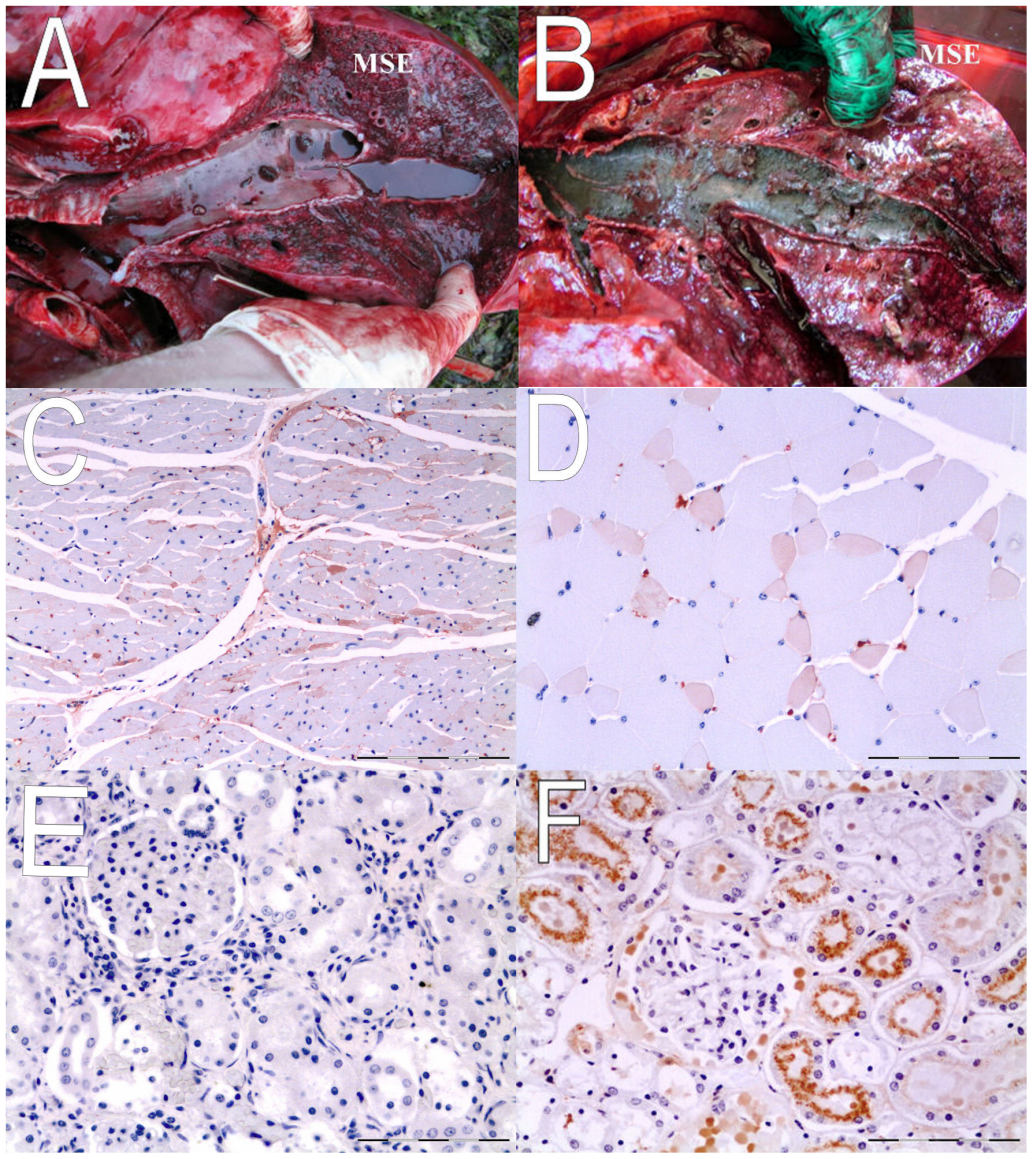
Gross pathology (lung) and anti-fibrinogen immuno-histochemistry (IHC) of cardiac and skeletal muscle and anti-myoglobin IHC of kidney. A (lung) – watery fluid and small clumps of mud in bronchi (gross); B (lung) – copious muddy substrate in bronchi (gross); C – acute myocardial degeneration – cardiac muscle (anti-fibrinogen IHC); Bar  = 50 µm; D – rhabdomyolysis – skeletal muscle (anti-fibrinogen IHC) Bar  = 50 µm; E – live stranded common dolphin from MSE negative for myoglobinuria (anti-myoglobin IHC) Bar  = 50 µm; F – myoglobinuria in live stranded striped dolphin from Canary Islands, Spain (positive control – anti-myoglobin (IHC); Bar  = 50 µm.

Eleven dolphins had moderate to copious quantities of muddy substrate in the trachea and bronchi macroscopically (Table 2; Fig. 4A). Particulate matter (mud) and plant material could be seen grossly and microscopically in many bronchial, bronchiolar and alveolar spaces (Fig. 4B). Another three dolphins had traces of particulate matter visible microscopically within bronchiolar and alveolar spaces (Table 2). Acute vascular congestion of internal organs and acute cortical tubular necrosis (kidney) were common microscopic findings. Widespread acute myocardial degeneration (heart muscle) (Fig. 4C) and acute rhabdomyolysis (skeletal muscle) (Fig. 4D) were confirmed by anti-fibrinogen immunohistochemistry. Anti-myoglobin antibodies demonstrated only minimal to absent myoglobin urinary casts in formalin-fixed kidney samples of 26/26 (100%) of dolphins examined (Fig. 4E) when compared to the positive control material (Fig. 4F). There was no gross or microscopic evidence of adrenal enlargement in any of the 26 dolphins necropsied. Four out of 26 adrenal glands were grossly normal but too autolysed for histopathology. Of the other 22, 12/22 (54.5%) were markedly congested (mainly within the inner adrenal cortex), 9/22 (40.9%) had acute haemorrhages within the inner adrenal cortex and 2/22 (9.0%) had haemorrhages in the adrenal capsule.

Gross and microscopic examinations of the cerebrum, cerebellum and spinal cord failed to detect inflammatory or infectious processes in any of the animals examined (n = 26) (**Table** **2**). A small number of gas bubbles were seen in the mesenteric veins of some fresh carcasses but other tissues were generally free of macroscopic or microscopic bubbles. Intravascular bubbles were seen in some of the more-decomposed carcasses, but these were considered to be consistent with *post-mortem* change rather than *ante-mortem* gas embolism. Lung and lymph node samples were negative for the presence of fat emboli in all 26 dolphins examined.

### Gross and histopathological examinations (Tympanoperiotic complexes)

**Figure 5:**
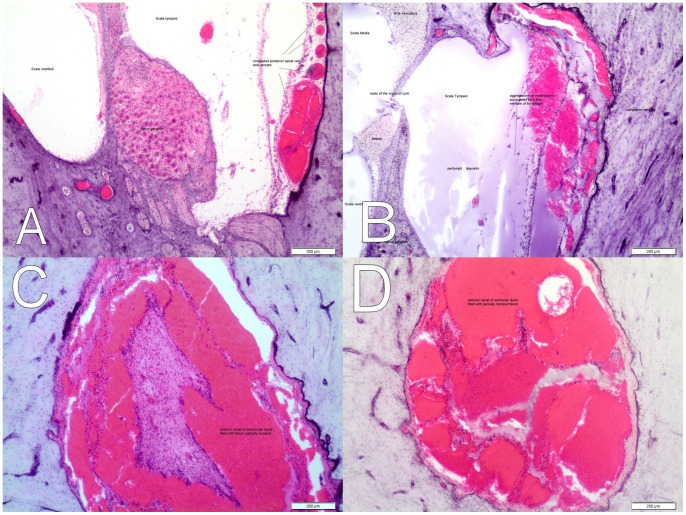
Histopathology of the tympanoperiotic complex. A – congestion of posterior spiral vein and venule (ref: SW2008/94.14) Bar  = 200 µm; B – perilymph deposits, vascular congestion and aggregates of erythrocytes surrounded by a fine network of fibroblasts (ref: SW2008/10) Bar  = 200 µm; C – anterior canal of semicircular ducts filled with blood (ref: SW2008/94.2) Bar  = 200 µm; D – anterior canal of semi-lunar ducts filled with blood (ref: SW2008/94.10) Bar  = 200 µm.

The ears (tympanoperiotic complex) appeared to be grossly normal in all cases, apart from two dolphins that had silt lining the outside of the tympanic bullae. Parasites were not seen macroscopically in all auditory complexes but were seen in 1/4 animals examined microscopically (SW2008/94.2 left). Only six dolphin ear specimens were examined microscopically and these consisted of the complete tympano-periotic complexes of SW2008/94.2 (left ear only); SW2008/94.10 (left and right ear), SW2008/94.14 (left and right ear) and SW2008/94.12 (periotic bone only) (Table 3) (Fig. 5A–D). Parts of the auditory tube were visible only in the left ear of SW2008/94.2 and the right ear of SW2008/94.10. The corpus cavernosum tympani was present in all investigated ears, apart from the left ear of SW2008/94.10. The results of the microscopic examinations of the ears in these cases are summarised (Table 3) and illustrated (Fig. 5A–D).

**Table 3:** Pathological and other data for common dolphin ears examined microscopically.

Case number	State of decomposition	Microscopic pathological findings
SW2008/94.2 (left)	moderate to poor	The ossicles were intact and there was severe haemorrhage in the semicircular canals and haemorrhage in the tympanic cavity (Fig 5C). The bones of the tympano-periotic complex show mild age related changes.
SW2008/94.10 (left and right)	moderate	The ossicles were intact and the caudal parts of the left medial lobe of the tympanic bone were fractured (extraction artifact). There was bilateral moderate haemorrhage in the basal turn of the scala tympani and moderate to severe haemorrhage in the tympanic cavity and the left anterior semicircular canals (Fig 5B and 5D).
SW2008/94.12 (right cochlea only)	moderate	There was a severe haemorrhage in the basal turn of the scala tympani and the anterior semicircular canal and moderate haemorrhage present in the tympanic cavity. The tympanic bone showed moderate age related changes.
SW2008/94.14 (left and right)	moderate to poor	The ears were in a moderate to poor condition. There was congestion of the posterior spiral vein and venule (Fig 5A). There was a bilateral mild, multifocal, acute otitis media associated with foreign plant material. The ossicles were intact. There was a bilateral haemorrhage in the basal turns of the scala tympani, the tympanic cavity and the anterior semicircular canals.

### Bacteriological findings

Bacteriological findings were unremarkable and, microscopically, there was no evidence of host reaction to bacterial organisms, indicating only probable *post-mortem* colonisation of tissues. The bacterium *Brucella ceti*, which has been associated with lesions in cetaceans [18], was isolated from a grossly and microscopically normal sample of testis from a single juvenile dolphin, but this bacterium was not isolated from any other tissues from this animal.

### Morbillivirus detection

There was no evidence of morbillivirus RNA in lung (n = 26) or brain (n = 22) samples tested (**Table** **2**).

### Chemical and algal toxin detection

Analyses of frozen blubber samples from five sexually mature lactating females, one immature female and one immature male dolphin were determined with mean levels of 10.7 mg/kg lipid weight (range: 2.80–30.6) for the sum of 25 chlorinated biphenyls (Σ25CBs). Mean concentrations (mg/kg lipid weight) were 0.17 (range: 0.05–0.45) for *p,p*′-DDT; 0.86 (0.13–2.86) for *p,p*′-DDE; 0.09 (0.02–0.23) for *p,p*′-TDE, 0.01 (0.011–0.014) for alpha-hexachlorocyclohexane; <0.01 (all cases were below limit of analytical detection) for gamma-hexachlorocyclohexane; 0.02 (0.01–0.04) for dieldrin and 0.05 (0.01–0.04) for hexachlorobenzene. Analyses of frozen liver tissues from the same adult animals determined mean concentrations (in mg/kg wet weight) of 31.9 (range: 9.20–53.0) for Hg; 0.29 (0.10–0.50) for Cd; 0.03 (0.02–0.03) for Pb; 0.08 mg/kg (0.07–0.11) for Cr; 3.14 (2.70–3.80) for Mn; 168.1 (109.0–221.0) for Fe; 0.07 (0.05–0.08) for Ni; 6.40 (5.40–7.90) for Cu; 49.6 (40.0–90.0) for Zn; 0.36 (0.28–0.43) for As; 13.8 (5.40–22.0) for Se and 1.41 (0.72–2.60) for Ag. The molar Hg:Se ratio was less than 1.0 in all common dolphin livers examined. The mean concentration of the summed hepatic concentrations of monobutyltin, dibutyltin and tributyltin was 0.03 (range: 0.021–0.054) mg/kg wet weight.

**Figure 6:**
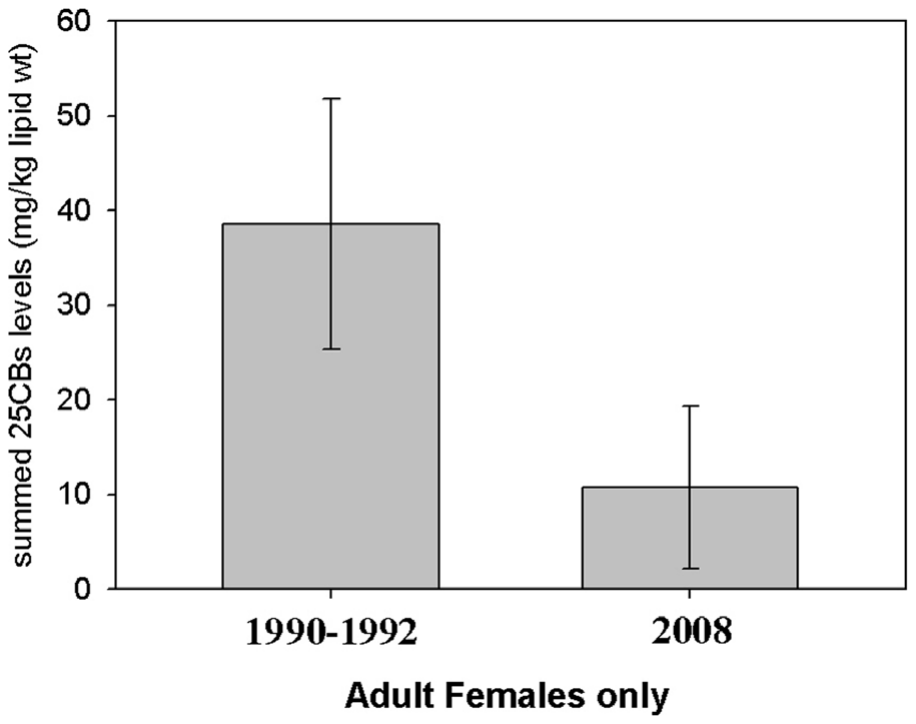
Mean summed 25CBs levels in UK-stranded adult female common dolphins from 1990–1992 (n = 8) and from the MSE in 2008 (n = 6). Error bar = 2 S.E.

Using available toxicological data from adult female UK-stranded common dolphins in 1990–1992 for comparison [12], [29], mean levels of all organochlorine contaminants examined (including Σ25CBs; **Figure** **6**) were lower in the five 2008 MSE adult female common dolphins which were examined for contaminants. The 1990–92 sample was composed of five mature females, including one female with a Σ25CBs of 95.6 mg/kg lipid weight. Mean hepatic concentrations of all butyltins and Cr, Ni, Cu, Zn, As, Se, Ag, Cd, Hg and Pb were again lower in the adult (male and female) dolphins from the MSE in 2008 than in the adults from the 1990–1992 group. No toxicological data were available for comparison from the 1990–1992 period for Mn, Fe or Ag.

Analyses of frozen liver tissues from the immature and mature animals selected for analysis (six female, one male) were negative for the presence of a suite of algal toxins including neurotoxic compounds e.g. domoic acid and saxitoxin, and also for toxins known to cause gastrointestinal disturbance including okadaic acid and dinophysis toxins (**Table** **2**).

### Disturbance by marine noise

**Figure 7:**
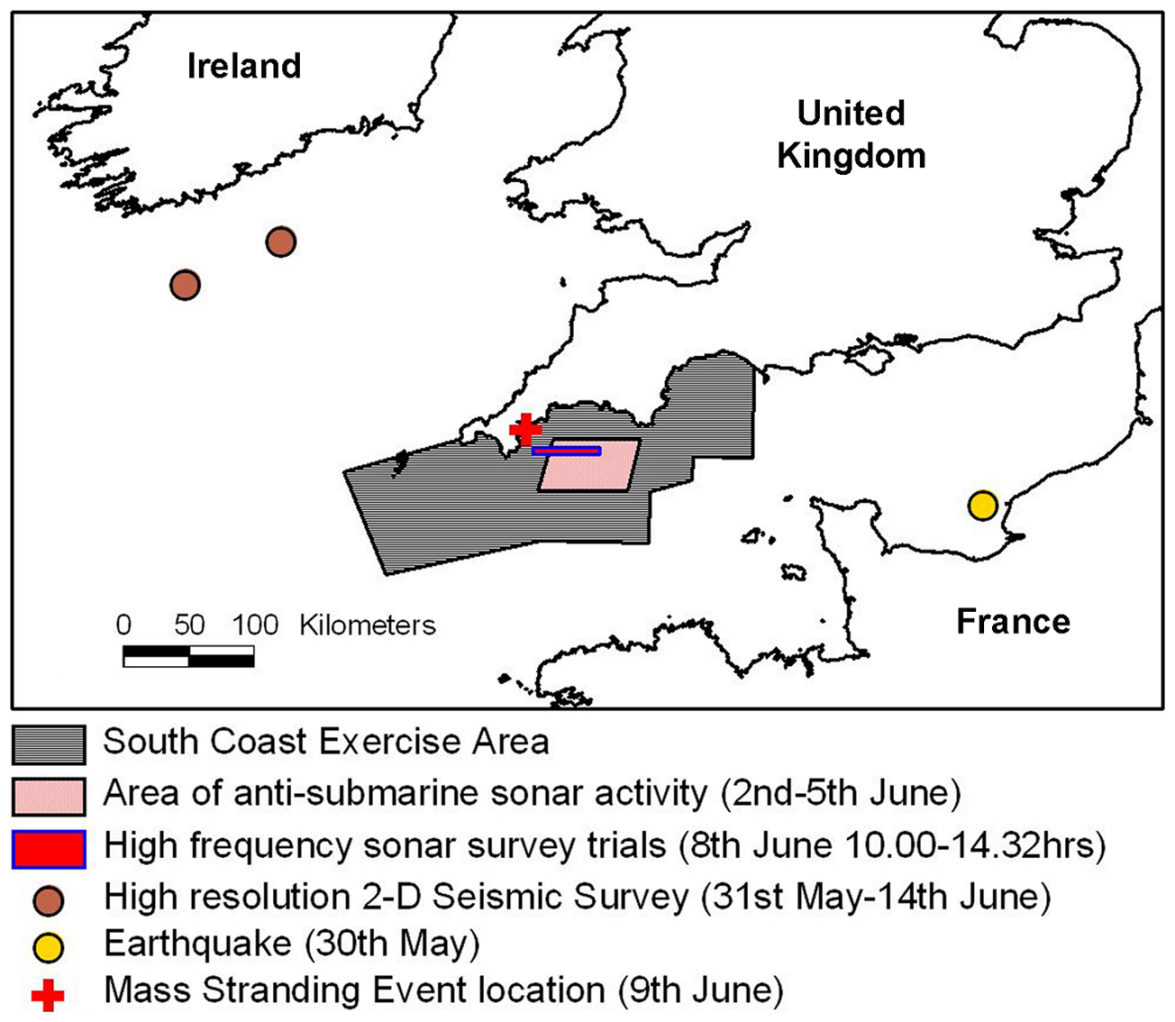
Distribution of high-intensity acoustic activities nearest to stranding location.

The hypothesis that marine noise coincident with the strandings could have been responsible for the MSE was considered. No acoustic recordings from the time of the stranding were available to allow assessment of sound levels. Information on high-intensity acoustic activities in the general vicinity of the MSE showed the nearest earthquake to the stranding date and location was recorded off the coast of northern France near Le Havre (approximately 330 km from Cornwall), 2.8 ML magnitude recorded at 10 metres below seabed on 30 May 2008 (source: *European-Mediterranean Seismological Centre*) (**Figure** **7**). The UK Department of Energy and Climate Change (DECC) confirmed that no geophysical surveys, including surveys involving seismic methodologies, were licensed to take place in the English Channel or Southwest Approaches, or in any adjacent sea area, either immediately prior to or during the stranding event. A research vessel (*RV Celtic Explorer*) was conducting a high-resolution 2-D seismic survey in the Celtic Sea south of Cork, Republic of Ireland, using a relatively small sized array of 150 cubic inches (commonly arrays can exceed 3000 cu inches for 2-D surveys), from 31 May until 14 June at a distance from the MSE of over 200 km with intervening land (data: *Marine Institute*) (**Figure** **7**). Falmouth Harbour Authority records show no unusual commercial shipping movements in or out of the harbour prior to the MSE. No record of movements of small pleasure craft or the use of non-military sonars exists, but no particular large-scale events likely to cause significant noise inputs (e.g. a powerboat race) occurred in the area during the relevant period. A commercial dockyard exists in Falmouth Harbour within the estuary. The dockyard is mainly a dry dock and the management reported no unusual activity around this time.

Naval exercises involving up to 20 Royal Navy surface and submarine vessels and 11 vessels from seven other countries (Holland, France, Germany, USA, Belgium, Chile and Denmark) was conducted in the South Coast Exercise Area off the southern coastline of Cornwall, Devon and Dorset from 1–9 June, with peak activity on 4–5 June (see **Figures** **7** **and** **8**) (data: UK Ministry of Defence). Routine naval exercises using live munitions, helicopters and fixed-wing aircraft and mid-frequency sonars (deployed from surface ships and helicopters) in the South Coast Exercise Area – which extends from Dorset to Cornwall – occur on about 46 weeks of each year (data: UK MoD). The MoD provided records of activity of all MoD vessels and foreign naval vessels, including associated maritime aircraft, operating under UK Operational Control in the South West and central Channel regions during the 7 days preceding the MSE (1–9 June 2008). The MoD does not hold records of the movement of other vessels in these areas, either UK or foreign registered nor does it have records of the activities of foreign naval vessels not under UK Operational Control. Foreign naval vessels do have the right of innocent passage through UK Territorial Waters but innocent passage would preclude operation of sensors such as active sonar which are not required for navigation (data: UK Ministry of Defence).

**Figure 8:**
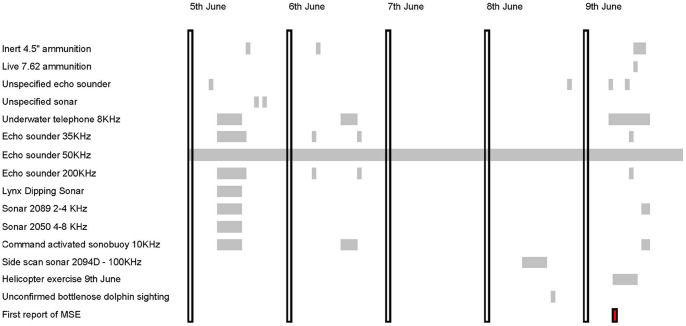
Temporal distribution (5-9 June 2008) of naval acoustic activities and possible bottlenose dolphin (*Tursiops truncatus*) sighting in western part of South Coast Exercise Area.

During the period from 1 June until the afternoon of 9 June, intermittent and occasional acoustic outputs from naval vessels were reported, including the use of standard echosounders (35/50/200 kHz), other sonars, acoustic modems, autonomous sonobuoys, and the firing of live, inert and blank ammunition, including a single live Seawolf missile (data: UK Ministry of Defence). Antisubmarine warfare (ASW) activities using mid-frequency sonars (2–4 kHz and 5–8 kHz) were conducted in the areas Foxtrot, Golf and Hotel (Admiralty Chart 442, *UK Hydrographic Office*) at least 45–50 km from the stranding locations in Falmouth Bay (**Figure** **7**) up to 6 June, some 60 hours before the discovery of the stranded dolphins. On 8 June, a high frequency side-scan sonar trial (100 kHz) was conducted in an area approximately 15 and 50 km from the location of the MSE between 10∶00–14∶32 hrs (**Figure** **7**). A submarine was operating in the South Coast Exercise Area, including Falmouth Bay, on the 8 June and from 0900–1500 hrs on 9 June used an echo sounder (at 50 KHz) and passive sonar. A low-power (70 Watts) short range underwater telephone (8 kHz) was scheduled to be used from 08∶00 hrs on the morning of 9 June (**Figure** **8**) but this device was only used in the Plymouth area and after the dolphins had first started to strand at Porth Creek (data: UK Ministry of Defence). The unspecified echo-sounder deployed on 9 June (**Figure** **8**) was a standard navigational system deployed on a troop carrier (RNLN Amphibious Ship *Johan De Witte*) in the vicinity of Plymouth.

A helicopter flying exercise (not anti-submarine warfare) was scheduled to take place between 08∶00–13∶30 hrs on 9 June in the Falmouth Bay (North), Falmouth Bay and Mounts Bay exercise areas. The entire Falmouth Bay area on 9 June was allocated to RNAS Culdrose Squadrons (Royal Navy helicopter base), but the first flight reported to leave RNAS Culdrose was at 08∶58 hrs on 9 June (37 minutes after the MSE was first reported to Falmouth Coastguard). This helicopter returned to RNAS Culdrose at 09∶31 hrs and the first incoming flight from RAF Netheravon landed at RNAS Culdrose at 09∶17 hrs (around one hour after the discovery of the MSE) and did not use the Falmouth Bay area. There were no helicopter flights from UK or non-UK naval ships reported in the Falmouth Bay region immediately prior to the onset of the MSE and no sonar of any type was deployed by the helicopters (data: UK Ministry of Defence). Naval activities incorporating anti-submarine mid-frequency dipping sonars and firing of inert rounds resumed (after a 3–4 day period of abstinence) on the afternoon of 9 June after the MSE (data: UK Ministry of Defence). The airspace in the vicinity of St Mawes is uncontrolled and available to all civilian aircraft and such aircraft do not need to file a flight plan or utilise an air traffic service (data: Civil Aviation Authority) and so the possibility that civil aircraft were in the vicinity cannot be discounted.

### Disturbance by other cetacean species

Marine mammal top predators like bottlenose dolphins (*Tursiops truncatus*) and killer whales (*Orcinus orca*) are known to attack and kill or, in the case of killer whale, predate on other marine mammal species [37], [38], [39]. The nearest reported sightings of killer whales to the MSE were in waters off west Wales on 1 June 2008 and off northern Scotland on 7–8 June 2008 (data: Janet Baxter *pers com.*; *Seawatch Foundation*). An unconfirmed report of two bottlenose dolphins in Falmouth Bay was made by naval Marine Mammal Observers (MMOs) at 17∶22 hrs on 8 June. Other confirmed and unconfirmed reports of bottlenose dolphins were made within 25km of Falmouth Bay both before and after the MSE (**Table 1**). These sightings are consistent with the presence of a small group of inshore bottlenose dolphins that is known to have been continuously resident around the southwest peninsula, including the Falmouth Bay area, since 1991 (data: Cornwall Wildlife Trust).

### Geographical and other factors

**Figure 9:**
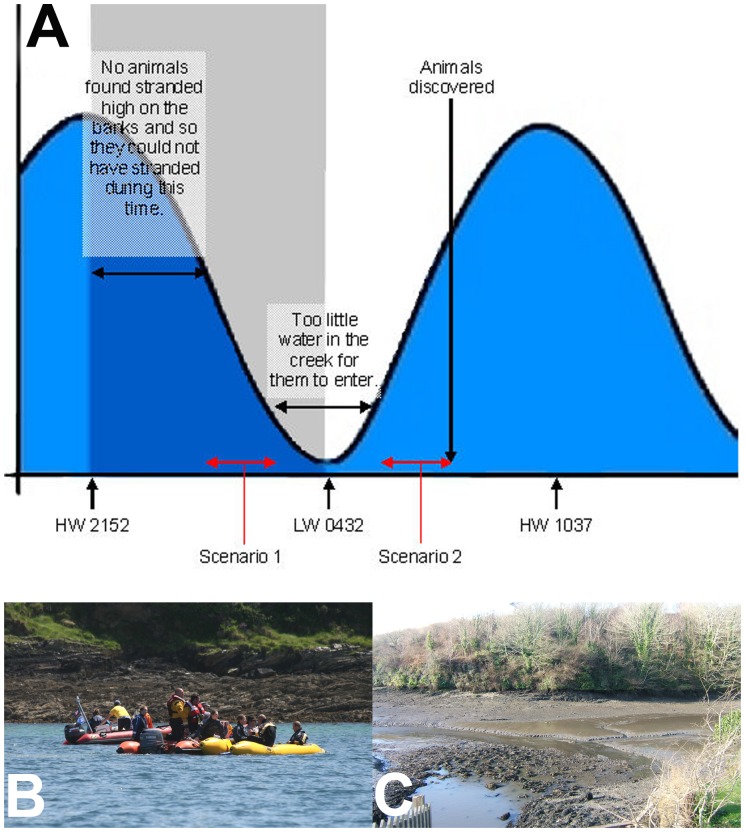
Tidal cycle at Froe (Porth Creek) from Sunday evening (8 June) to Monday afternoon (9 June) in 2008. (A) There are two scenarios for when the dolphins may have entered (Scenario 1 on an ebbing tide and Scenario 2 on a rising tide). The pathological evidence of aspiration of mud/seawater in the lungs of 11/26 dolphins was most consistent with death occurring on a rising tide (Scenario 2). Image courtesy of CWT Marine Strandings Network (B) Photo of Froe (Porth Creek) at/near high water (photo credit: C. Curtis). (C) Photo of Froe (Porth Creek) at low water showing gently shelving bank in the distance (photo credit: D. Wallis).

The strandings were in shallow, tree-lined, narrow tidal creeks with muddy substrates where common dolphins are not normally seen. Only two records among 7,700 cetacean sightings reports held by the Cornwall Wildlife Trust were from this area and involved two common dolphins that stranded alive at different times in different tributaries of the Fal. Common dolphins are frequently present in the open bay, although usually two kilometres or more from the shore (data: Ray Dennis *pers com.*/Cornwall Wildlife Trust Marine Sightings Database). There were no reports from local fisheries of unusual bait fish distribution or fish die-offs in the Falmouth Bay region (data: Cornwall Sea Fisheries Committee). The tidal cycle for Porth Creek where most of the live and dead dolphins were found is shown in **Figure 9**. The 8–9 June period was close to the peak of the spring tides (data: UK Met Office).

## Discussion

The purpose of this investigation was to conduct rigorous necropsies and additional diagnostic tests on all 26 dead dolphins to try to determine the main cause, or identify a number of likely causal factors, of the Falmouth common dolphin MSE on 9 June 2008. Falmouth Marine Coastguard Agency telephone records show that the first call reporting a common dolphin in Porth Creek was received at 08∶21 hrs. More live and dead dolphins were discovered as the morning progressed but there remained some uncertainty about the actual onset of the MSE in the absence of any earlier eyewitnesses prior to approximately 08∶00–08.15 [17], [40]. It therefore fell under the remit of the investigation not just to identify likely causal factors, but to try to more accurately determine the most likely time of onset of the MSE.

All necropsied common dolphins in this MSE were either sexually immature individuals of both sex or sexually mature lactating females. Sex and/or length data are not available for individuals that were successfully refloated. The calving/mating period for common dolphins in the North-east Atlantic ranges from May to September inclusive [25], and although three of the mature females were heavily lactating, and one showed evidence of recent gravidity, no newborn/neonates were observed in the necropsied sample. All immature necropsied dolphins ranged between 1.9 and 9 years in age. A lactation period of 10.35 months has been proposed for this population [13] and both milk and solid food have been observed in the stomachs of calves between three and six months of age [41]. This all suggests that newborns/neonates would have been part of the original group.

Eleven dolphins had moderate to copious quantities of muddy substrate in the trachea and bronchi indicating asphyxiation due to inhalation of mud and/or water (drowning) following stranding alive as the most probable cause of death of these animals. All dolphins in this MSE appeared to be in good nutritional condition and showed little evidence of acute physical injury or disease that might have precipitated the MSE. A number of potential causes of the MSE can therefore be either eliminated or considered highly unlikely. These include by-catch since no acute traumatic external or internal lesions consistent with by-catch [11], [12] were found in any of the dolphins and because all dead dolphins appeared to have stranded alive. The absence of stomach contents in the examined dolphins in this MSE probably rules out a hazardous pursuit of prey as the immediate cause of this event. Often fatal attacks by bottlenose dolphins on harbour porpoises [37], [38] and occasionally on other species including common dolphins [42], [43] have been reported in the British Isles and elsewhere, but these typically produce distinctive external and internal injuries and none of these characteristic lesions were seen in any of the dead or surviving common dolphins or in video footage of the live animals. A small group of bottlenose dolphins is known to have been continuously resident around the southwest peninsula, including this area, since 1991 (data: Cornwall Wildlife Trust), so the coincidence of the two species is not unusual and cetacean MSEs have not been causally linked to negative interactions with bottlenose dolphins.

A common dolphin mass mortality event in 1994 in the Black Sea [44] was linked to distemper caused by cetacean morbillivirus infection and occurred over a much longer period of time and a wider geographic area than this event. No lesions consistent with distemper were found in the dolphins in this MSE and all molecular tests for morbillivirus infection were negative. Brucellosis (associated with *Brucella ceti*) has been associated with high polychlorinated biphenyl (PCB) exposure in bottlenose dolphins that stranded in Cornwall in recent years [45]. Meningoencephalitis associated with *Brucella ceti* infection was also reported in a striped dolphin that stranded alive in Cornwall [46]. However, the *Brucella ceti* isolated from the testis of one common dolphin in this MSE was from grossly and microscopically normal tissue and therefore considered incidental to the dolphin's cause of death. A longstanding hypothesis of a “sick lead animal” that may lead other cetaceans in its social group to strand [8] also appears unlikely here both because no such individual was found among the dead (although one unconfirmed dead dolphin was reported but not retrieved). Analytical tests of tissue samples showed that they were free of harmful algal toxins and the levels of organochlorines (polychlorinated biphenyls and pesticides such as DDT), trace metals and butyltins were at relatively low levels and generally lower than levels recorded in by-caught common dolphins stranded in south-west England in 1990–92. However it should be noted that all mature females in the MSE sample were actively lactating and so would be offloading most of their body PCB burden.

As the very earliest stages of decomposition were already present in all of the dolphins at the time of necropsy, if present, subtle inner-ear injuries potentially related to direct high-intensity acoustic exposure [21] may have been difficult to detect histopathologically. In 4/4 cases examined microscopically, however, examination of the auditory apparatus showed significant haemorrhages in the tympanic cavity and scala tympani including one dolphin with older haemorrhage characterised by red blood cells within a fine web of fibroblasts across the cavity of the scala tympani or the perilymph spaces of a semicircular canal. The web of fibroblasts is found in all cetaceans around the apex of the cochlea and may have an anatomical rather than pathological function. One dolphin also had a bilateral, mild, multifocal acute otitis media that was not considered severe enough to have had a causal role in the MSE. The significance of the haemorrhages in the context of the cause of the MSE is less clear. Although haemorrhages have been purported to be caused by auditory trauma linked to high-intensity acoustic exposure [22], [47] haemorrhages in a range of cetacean tissues are common in many stranding situations and different causes of death, including by-catch and other forms of trauma, stranding alive and a range of infectious diseases [11], [37], [48]. Better baseline data on cetacean auditory anatomy and pathology is needed to help interpret the significance of any lesions in the auditory apparatus of cetaceans in single and mass stranding events.

Overall, the pathological investigations undertaken indicate that the animals were healthy until they stranded, but provide no further indicators as to the likely cause. The possibility exists that the MSE occurred due to some intrinsic error of “navigation” within a social group of dolphins. The 8–9 June period was close to the peak of the spring tides and this could theoretically have part-influenced the onset of a MSE due to navigational error via the occurrence of rapidly receding tides in the coastal creeks where the MSE occurred. Spring tides are part of normal tidal cycles, however, and there is no established correlation between spring tides and cetacean MSEs. One study has modelled coastlines where low frequency cetacean echolocation signals might be distorted by purely geometric effects [6] but this was considered unlikely to explain this MSE because Falmouth Bay is not a “hotspot” for cetacean MSEs and common dolphin echolocation is at frequencies far higher than those that might fit a model for larger whales using lower frequencies [6].

For centuries humans have made sounds to induce mass strandings of otherwise healthy small cetaceans in “drive fisheries” for food [49]. High intensity acoustic energy sources were therefore considered as a potential trigger of the MSE. The seismic survey by the *RV Celtic Explorer* appears to have been too distant, and the earthquake occurred too long before the event for these to be likely causes. Nearby dockyard activity was reported to be normal and is therefore unlikely to have attracted animals into its proximity. Military acoustic sources (particularly mid-frequency anti-submarine sonars) have been causally linked with cetacean MSEs predominantly involving beaked whales [23], [50], [51], [52], [53], [54]. It has been proposed that beaked whales (and possibly other cetaceans) may show hazardous behavioural changes in response to some sonar frequencies, potentially leading to nitrogen supersaturation and risk of gas and fat embolism similar to decompression sickness in humans [23], [54], [55], [56]. Cases of acute and chronic gas embolism have occasionally been found in the UK, including in common dolphins stranded in SW England [11], [54], [56] but no evidence of significant gas or fat emboli were found in any of the 26 examined dolphins. Neither were any lesions found consistent with intense sound or shock wave exposure (reviewed in [21], [51], [57], [58], [59], with the possible exception of the haemorrhages in the auditory apparatus (middle and inner ears). Acute physical trauma (such as a boat impact) could have theoretically caused the haemorrhages in the ears but such boat impacts are associated with marked subcutaneous and other non-auditory bruising and/or other injuries [10], [11], but none were found. Apart from some form of acoustic trauma, the haemorrhages in the ears may be caused by hemodynamic changes in vascular perfusion associated with “stranding stress” and hypertension linked to progressive cardiovascular and pulmonary collapse following stranding alive.

It is theoretically possible that a MSE can be acoustically-mediated via the induction of a panic/flight behavioural response(s) that directly leads to stranding and death in the absence of gas or fat embolism or other “acoustically-induced” lesions. Such a mechanism may underpin this common dolphin MSE. However, such a phenomenon is difficult to demonstrate conclusively because there are no analytical or forensic tests available to qualitatively or quantitatively assess the history of acoustic exposure in a dead animal and, by definition, these types of MSEs would not present any lesions (e.g. gas embolism) suggestive of acoustic exposure. One well studied non-beaked whale cetacean MSE causally linked to high-intensity anthropogenic sound was the “near-MSE” of melon-headed whales (*Peponocephala electra*) in Hawaii in 2004, that most likely resulted from mid-frequency sonar deployed by the U.S. Navy where a group of normally pelagic animals entered a shallow bay very soon after active mid-frequency sonars were deployed nearby. They exhibited “milling” behaviour and remained (even after the sonar was turned off) until humans finally herded them out of the bay [60]. A behavioural review of this species strengthened the case that mid-frequency sonar played a major role in this near-MSE [61]. Near-shore “milling” behaviours were seen in several small groups of common dolphins around the time that dolphins were first reported at Place and Porth Creek around 08.15 and later in the inner part of Falmouth Harbour and Gillan Creek. This milling behaviour, together with the flight/panic behaviours seen in other individuals is therefore highly consistent with acoustic disturbance [60], [61] although milling behavior is a common finding in other common dolphin mass stranding hotspots such as Cape Cod, USA where no linkage between acoustic stressors and mass stranding events has been shown [1].

Although not always causally linked, a growing number of cetacean MSEs had spatial and temporal associations with naval activities in recent years. These include 145 long-finned pilot whales (*Globicephala melas*) which stranded and died in a series of three events in the Marion Bay region in the Southeast of Tasmania, 25–27 October 2005 [62]; a mixed-species mass stranding event involving 33 short-finned pilot whales (*Globicephala macrorhynchus*), two dwarf sperm whales (*Kogia sima*) and one minke whale (*Balaenoptera acutorostrata*) in North Carolina on 15–16 January 2005 [63] and a mass stranding of four Cuvier's beaked whales in Almeria, Spain in January 2006 [64]. A mixed-species unusual mortality event involving multiple deep-diving cetacean species and 23 individual cetaceans in Taiwan between 19 July and 13 August 2005 was spatially and temporally linked to naval exercises [65] and another nine beaked whale MSEs were causally associated with naval sonars or other high-intensity acoustic naval activities in the Mediterranean and Caribbean Seas [52]. Increased numbers of stranded harbour porpoises were associated with naval sonars in Washington State, USA from 02 May to 02 June 2003 [66] and increased numbers of stranded harbour porpoises (n = 85) (mainly bycatches) were spatio-temporally associated with an international naval exercise in Danish waters from 7–15 April in 2005 [67]. A mass mortality of four long-beaked common dolphins (*Delphinus capensis*) was caused by primary blast injuries (barotrauma) induced by naval activities (munitions disposal) in Southern California on 4 March 2011 [57]. Most recently, a mass stranding of long-finned pilot whales in Scotland in July 2011 was correlated in space and time with munitions disposal in northern Scotland, although the investigation into the cause of this MSE is currently ongoing (unpublished data).

Naval exercises or some other unknown anthropogenic or natural event could have caused the large group of 50–60 common dolphins to come unusually close to shore in the days leading up to this MSE. This large dolphin group was seen unusually close to shore in the 3–4 days leading up to the MSE and may have been the same group that subsequently mass stranded on 9 June. This MSE may even have been a “two-stage process” where a group of normally pelagic common dolphins entered a coastal bay (possibly to avoid a perceived acoustic threat) and then, after 3–4 days in/around Falmouth Bay, a second acoustic or other disturbance event occurred nearer to shore that caused them to subsequently strand *en masse*. Several consecutive days of anti-submarine warfare training using mid-frequency active sonars ended on 6 June, some 60 hours before the MSE was first reported. Based on available evidence, active mid-frequency sonars may have been a factor causing a large dolphin group to move closer to shore several days prior to the MSE, but were considered unlikely to have directly triggered the event on 9 June.

Similarly, high-frequency sonars were considered unlikely to have triggered the MSE. The towed high-frequency (100 kHz) side-scan sonar trial conducted at 10∶00–14∶32 hrs on 8 June was between 15–50 km from the location of the MSE and terminated approximately 18 hours before the MSE was discovered. Such high-frequency acoustic devices are commonplace in the marine environment, occurring on almost all vessels and, although high-frequency systems are often components of both naval exercises and seismic surveys, they have not been directly implicated (causally) in any cetacean MSE.

Establishing the precise timing of the onset of an MSE is critical to determining a potential cause, particularly if an immediate behavioural response to an anthropogenic acoustic event is suspected. Although in the 2008 MSE the first dolphin was reported at 08∶21 hrs on 9 June, the tide cycle would have allowed dolphins to enter Porth Creek much earlier (see **Figure 9a**). A period around the ebb tide was also considered inaccessible for stranding due to the lack of water. There are therefore two scenarios for when the dolphins could have entered Porth Creek, one on an ebbing tide and one on a rising tide (**Figure 9a–c**). Based on the times of high and low water, it is possible that the common dolphins entered the inner parts of Porth Creek (where they were first discovered) either after 06∶30–07∶00 hrs on 9 June or sometime shortly after 00∶00 hrs on 9 June. The period around two hours either side of low tide at 04∶32 would have prevented dolphins entering the creek between 02∶30–06∶30 (approx.) because the water level would be too shallow (see **Figure 9a–c**). All of the dead dolphins retrieved for necropsy in Porth Creek were found below high water level as the incoming tide was still rising. This is consistent with the dolphins not ever being in the creek at high tide (dead or alive) otherwise at least some might have been found stranded high on the bank at the high water level. It is therefore most likely that they entered sometime after 06∶30 hrs on 9 June on the incoming tide once the water was sufficiently deep for them to swim into the creek.

The MSE was close to spring tide but there is no scientific evidence linking cetacean MSEs with spring tides as causal factors. Furthermore, the dolphins in this MSE stranded on the incoming tide mid-way in the tide cycle – so there was neither a particularly high or low tide at the beginning of the MSE onset. The presence of water and mud in the lungs in 11 of the dead dolphins was consistent with them stranding alive on a rising (incoming) tide and drowning. Based on 20 years of necropsies of UK-stranded and by-caught cetaceans, water is rarely, if ever, found in the lungs of cetaceans that have died of asphyxiation in fishing nets (by-catch), demonstrating that water is not aspirated during the by-catch process and that the blowhole probably remains closed after death preventing entry of seawater [11], [12]. In our experience, water in the lungs is usually only found in necropsies of cetaceans that have stranded alive and where an incoming tide eventually submerges the blowhole resulting in drowning by terminal aspiration of seawater (P.D. Jepson, *pers obs*). A similar mechanism is likely to explain the agonal inspiration of water and mud found in the lungs of 11/26 dead dolphins in this MSE.

Diseases such as stress cardiomyopathy [68], capture myopathy [69] and contraction band necrosis [70] may cause rapid death in mammals exposed to extremely stressful events (such as capture, violent assault or beach stranding), with cetaceans appearing particularly vulnerable to these conditions [71]. Evidence of rapid death was universally present in this MSE, including acute congestion of internal organs, widespread acute myocardial degeneration (heart muscle), acute rhabdomyolysis (skeletal muscle) and acute cortical tubular necrosis (kidney). These lesions are consistent with acute cardiovascular failure and stranding (capture) myopathy associated with beach stranding [24]. However, these acute changes occurred largely in the absence of renal myoglobin urinary casts (myoglobinuric nephrosis). Extensive myoglobinuric nephrosis has been described in a striped dolphin (*Stenella coeruleoalba*) that was beached alive for a prolonged period before death [24]. A study of humans with severe muscle injury also showed development of myoglobin urinary casts in kidneys within 1 hour between muscle injury and death [72]. The absence of myoglobin urinary casts is, therefore, suggestive of fairly rapid death without a prolonged period of being beached alive. If dolphins had entered the Creek on the ebb tide then stranded overnight and drowned on the incoming tide we would have expected to see myoglobin urinary casts in the kidneys. Conversely, if dolphins had entered the creek on the ebb tide and quickly died, there would not have been enough seawater in the Creek to have drowned 12 of them. Collectively, these findings are most consistent with a relatively rapid death after entering Porth Creek on the incoming tide on the morning of 9 June. This technique of combining inhalation of seawater (drowning), the tidal cycle and the presence or absence of myoglobin urinary casts in kidneys could be used to help determine the precise timing of stranding in future MSE events.

If the dolphins entered Porth Creek sometime after 06∶30 hrs and before 08∶21 hrs on Monday 9 June, the closest naval activity in time and space was the helicopter flying exercise scheduled for Mounts Bay, Falmouth Bay North and Falmouth Bay regions between 08∶00 and 13∶30 on Monday 9 June. On 9 June, all naval helicopter flights in the Falmouth Bay region were allocated to RNAS Culdrose Squadrons. The first flight recorded to leave RNAS Culdrose (nearest Royal Navy helicopter base) was at 08∶58 hrs on 9 June (37 minutes after the MSE was reported) and the first incoming flight landed at 09∶17 hrs (around one hour after the first MSE report) so these flights, or any subsequent ones that day, could not have been the immediate trigger of the MSE. However, naval helicopter activity, or some other unknown factor(s), may have caused the apparently agitated/flight response in the group of dolphins later seen entering the inner part of Falmouth Harbour around 09∶30 hrs (approx 80–90 mins after the initial discovery of the MSE). Naval exercises using live munitions, helicopters and fixed-wing aircraft and mid-frequency sonars (deployed from surface ships and helicopters) in the South Coast Exercise Area – which extends from Dorset to Cornwall – occur on about 46 weeks of each year (data: UK MoD). In terms of potential disturbance to marine mammals, helicopters generally elicit greater behavioural responses in cetaceans than fixed wing aircraft (reviewed by [73], [74] and behavioural responses were generally greater in mothers with calves, in shallow waters, in situations where the initial observed behaviour was resting (and travelling in small delphinids) and when the aircraft flew at lower (<500 m) altitudes and at smaller lateral distances from aircraft to the animals (reviewed by [73], [74], [75], [76].

The number of detailed studies on the impacts of aircraft on cetaceans is relatively small and forms part of a wider overall knowledge gap on how cetaceans respond to anthropogenic noise [77]. Detailed long-term flight records (other than take-off and landing times) are not routinely kept for small civilian aircraft and so it is not possible to identify or exclude any low-flying civilian fixed-wing/rotary flights in the Falmouth Bay region on 8–9 June 2008 (data: Civil Aviation Authority). The low power/short range underwater telephone (8 kHz) system scheduled to operate on the morning of 9 June was not used until after the dolphins had started to strand, and was only used in the Plymouth area, so can largely be discounted as a potential cause. The “unspecified” echo sounder deployed on the morning of 9 June during and after the MSE onset was a standard navigational echo-sounder also deployed in the vicinity of Plymouth and is therefore highly unlikely to have triggered the MSE.

In relation to the military activity in the vicinity of the Falmouth MSE, the times and positions of all military sonar transmissions and aircraft flights were provided by the UK MoD under the Freedom of Information Act and no unclassified records of sonar transmission or naval flights were withheld. This provides some degree of confidence that all appropriate active sonar and other naval data has been obtained for the Falmouth MSE. The RN/MoD also provided a very large quantity of information in relation to the naval exercise – probably far more data than has been released in any previous cetacean MSE. Concerns have been raised within the scientific community about the veracity of acoustic information provided by military sources in relation to other cetacean MSEs, given that the military is usually the only source of data on naval acoustic activities in most of these events (e.g. [53], [58]). For example, the occurrence of an entire North Atlantic Treaty Organisation (NATO) naval exercise that was coincident with a Cuvier's beaked whale MSE on the west coast of Greece in October 1997 was withheld by NATO for over a decade and only relatively recently reported [52].

The Royal Navy uses a range of measures to mitigate potential impacts on cetaceans and other marine mammals including “soft starts” (the gradually progressive ramping up of active sonar source levels to allow cetaceans to move away from the vessel conducting the exercise), use of trained marine mammal observers and reduction of sonar source levels (i.e. power) when cetaceans are sighted close to a vessel operating sonar transmissions (data: UK MoD). The naval mitigation of potential impacts of helicopters and fixed-wing aircraft includes maintaining a 500 m minimum flight altitude, wherever practicable, if any cetaceans are seen on the surface (data: UK Ministry of Defence). The rarity of cetacean MSEs in this area indicates that either normal methods of mitigation of naval impacts on cetaceans in the naval exercise area are generally effective, or the conditions necessary for an acoustically-driven MSE have not previously occurred.

Following this MSE and recommendations from the organisations involved in the rescue of dolphins in the MSE, the UK Ministry of Defence initiated the Marine Underwater Sound Stakeholders Forum in the UK to regularly meet with all interested stakeholders (scientists, other Government Departments like Defra and a range of non-Governmental organisations) to discuss these issues in some detail. A direct line of communication was also established after the Falmouth MSE to facilitate rapid exchange of information between cetacean strandings/sightings organisations and Royal Navy Naval Command Headquarters to report groups of pelagic cetaceans seen unusually close to shore and potentially at increased risk of stranding. This was used to report a near-MSE of over 20 common dolphins in the Fal estuary in April 2009 that was seen 15 minutes after RN sonar trials were initiated in the region. The RN immediately modified the naval exercise (including use of active sonars) until the group of dolphins had returned to open sea several hours later. The need to alter training excercises due to the presence of dolphins has not subsequently occurred in this region. Such continual improvement of mitigation strategies by the military themselves is probably the best way to limit future environmental impacts of naval activities, including cetacean MSEs.

## Conclusions

In conclusion, a number of potential causes of the 2008 common dolphin MSE can be either excluded or considered highly unlikely. These include infectious diseases, gas embolism, fat embolism, boat strike, by-catch, attack from killer whales or bottlenose dolphins, feeding unusually close to shore immediately prior to stranding, ingestion of harmful algal toxins, abnormal weather/climatic/tidal conditions and high-intensity acoustic inputs from seismic airgun arrays, recreational craft and geological events. A group of 50–60 common dolphins was seen unusually close to shore in the days leading up to the MSE and may have been the group that subsequently mass stranded. It is therefore possible that the MSE was a “two-stage process” where a large group of normally pelagic dolphins initially entered a coastal bay (possibly to avoid a perceived acoustic threat) and then, after 3–4 days in/around Falmouth Bay, a second acoustic or other type of disturbance event occurred, causing them to strand *en masse*. The international naval activities that took place in very close temporal and spatial proximity to this MSE are the only established cause of cetacean MSEs which cannot be eliminated and is ultimately considered the most probable (but not definitive) cause.
